# Mechanical allodynia triggered by cold exposure in mice with the *Scn11a* p.R222S mutation: a novel model of drug therapy for neuropathic pain related to Na_V_1.9

**DOI:** 10.1007/s00210-020-01978-z

**Published:** 2020-09-24

**Authors:** Yosuke Matsubara, Hiroko Okuda, Kouji H. Harada, Shohab Youssefian, Akio Koizumi

**Affiliations:** 1 Tsumura Kampo Research Laboratories, Tsumura & Co., Ibaraki, Japan; 2 Laboratory of Molecular Biosciences, Graduate School of Medicine, Kyoto University, Kyoto, Japan; 3 Department of Health and Environmental Sciences, Graduate School of Medicine, Kyoto University, Kyoto, Japan; 4 Social Health Medicine Welfare Laboratory, Public Interest Incorporated Association Kyoto Hokenkai, Kyoto, Japan

**Keywords:** Na_V_1.9, Familial episodic limb pain, Neuropathic pain, Acetaminophen, Goshajinkigan, Kampo

## Abstract

Mutations within the *SCN11A* gene which encodes the voltage-gated sodium channel Na_V_1.9 mainly expressed in small fiber sensory neurons have been associated with neuropathic disorders; however, suitable medications have not been fully investigated. To develop drug therapies against Na_V_1.9-related neuropathic pain, we aimed to establish a novel model using mice carrying the *Scn11a* p.R222S mutation initially identified in patients with familial episodic limb pain that is characterized by paroxysmal pain induced by fatigue or bad weather conditions. We investigated the influence of cold exposure (4 °C, overnight) on the behavioral and biochemical phenotypes of *Scn11a* p.R222S mutant (R222S) and wild type C57BL/6N (WT) mice. We also tested the effects of acetaminophen (125, 250 mg/kg, perorally, p.o.) and traditional Japanese medicine, goshajinkigan (0.5 or 1.0 g/kg, p.o.), which are analgesic drugs prescribed to patients with neuropathic pain, in this model of cold-induced mechanical allodynia in R222S mice.

Cold-exposed R222S mice exhibited enhanced mechanical allodynia and thermal hypersensitivity compared with WT mice. The decrease of the mechanical withdrawal threshold in R222S mice was reversible 24 h after housing at room temperature. There was no significant change in the levels of interleukin-1β, interleukin-6, tumor necrosis factor-α, or interferon-γ in the plasma or spinal cords of WT and R222S mice after cold exposure. Both acetaminophen (250 mg/kg) and goshajinkigan (1.0 g/kg) significantly attenuated mechanical allodynia in R222S mice. The model of cold-induced mechanical allodynia in mice with the *Scn11a* p.R222S mutation is novel and useful for evaluating analgesic drugs for intractable neuropathies related to Na_V_1.9.

**Electronic supplementary material:**

The online version of this article (10.1007/s00210-020-01978-z) contains supplementary material, which is available to authorized users.

## Introduction

Voltage-gated sodium channels (Na_V_s) are a family of sodium ion channel proteins that play a crucial role in the electrical excitability of nerve systems and cardiac and skeletal muscles. Na_V_1.9, a tetrodotoxin-resistant subtype of Na_V_ encoded by the *SCN11A* gene, is mainly expressed in sensory neurons. Na_V_1.9 exhibits slow activation kinetics and plays a role in setting activation thresholds and membrane potentials (Wood et al. 2004; Bennett et al. 2019). Several recent studies have revealed that *SCN11A* mutations are associated with gain-of-function Na_V_1.9 phenotypes and are related to peripheral paresthesia, such as painless disorders (Leipold et al. 2013; Woods et al. 2015; Phatarakijnirund et al. 2016; King et al. 2017), painful small fiber neuropathies (Huang et al. 2014; Kleggetveit et al. 2016; Han et al. 2017), and familial episodic pain syndromes, including familial episodic limb pain that is clinically indicated as paroxysmal limb pain induced by fatigue, bad weather, or cold temperature during infancy to adolescence (Zhang et al. 2013; Leipold et al. 2015; Okuda et al. 2016; Kabata et al. 2018).

Previous studies have also described medications for painful disorders related to *SCN11A* mutations. Although lamotrigine alleviated the pain of patients carrying the *SCN11A* p.R222H mutation in one study (Han et al. 2017), it was ineffective against the pain of a patient with the same p.R222H mutation in another study (Tanaka et al. 2019). Gabapentin, a voltage-gated calcium channel blocker, frequently used for the treatment of neuropathy, failed to control the nociceptive pain of patients with the *SCN11A* p.R225C mutation (Castoro et al. 2018). Acetaminophen (AcAP) and cyclooxygenase-2 inhibitors, common analgesic agents, are often prescribed for patients with familial episodic pain syndromes (Okuda et al. 2016; Kabata et al. 2018), yet they were also unable to alleviate the pain symptoms. In Japan, goshajinkigan (GJG), a traditional Japanese medicine (Kampo), is prescribed to relieve low back pain, numbness, dysuria, pollakisuria, itch, blurred vision, and neuropathies (Tawata et al. 1994; Cascella and Muzio 2017). Several studies have revealed that GJG can improve neuropathic pain via multiple mechanisms, including the spinal activation of the kappa-opioid system (Suzuki et al. 1999a; Bahar et al. 2013; Higuchi et al. 2015). It is anticipated that potential candidates, including GJG, will be efficacious against Na_V_1.9-related painful disorders. However, due to the lack of appropriate in vivo models of the neuropathic disorders related to *SCN11A* mutations, it has not been possible to appropriately evaluate the efficacy of any candidate drug.

To develop effective drug therapies for painful disorders related to *SCN11A* mutations, in vivo evaluation is imperative. We previously reported that knock-in mice carrying the *Scn11a* gene R222S missense mutation, identical to mutations found in familial episodic limb pain patients, exhibited hyperexcitability of dorsal root ganglion (DRG) neurons compared with wild type (WT) mice. In addition, *Scn11a* p.R222S knock-in heterozygotes housed at normal temperature (23 ± 2 °C) tend to exhibit hypersensitivities to mechanical and thermal stimuli (Okuda et al. 2016). These *Scn11a* p.R222S mutant mice were expected to display pain behavior similar to familial episodic limb pain, such as cold-triggered pain. In this present study, we therefore first assessed the influence of cold exposure on the behavioral and biochemical properties of R222S mice to establish a novel in vivo model for Na_V_1.9 mutations and then attempted to detect the analgesic effects of GJG and AcAP on the cold-triggered pain behavior of the R222S mice.

## Materials and methods

### Animals

Animal experiment protocols were reviewed and approved by the Animal Care, Use and Ethics Committee at Kyoto University (approval no. MedKyo18523, March 8, 2018). We used 154 healthy 6–7-week-old male C57BL/6N WT mice (purchased from Japan SLC, Inc., Shizuoka, Japan) and *Scn11a* p.R222S knock-in heterozygote mice. Mice weighing 19–26 g were housed in plastic cages with ad libitum food and water under controlled temperature (24 ± 2 °C) and humidity (50 ± 10%) and a 14-h light/10-h dark cycle (lights on 07:00–21:00) prior to the start of experiments, according to the Animal Welfare Guidelines of Kyoto University. *Scn11a* p.R222S knock-in heterozygote mice used in experiments were the first litters obtained by interbreeding of male *Scn11a* p.R222S homozygote mice (generated in the previous study (Okuda et al. 2016)) and female C57BL/6N WT mice (Japan SLC). Male WT mice were used in experiments as negative control animals. At the end of experiments, mice were euthanized by isoflurane anesthesia following cervical dislocation or exsanguination.

### Cold exposure

Mice were housed in plastic cages with ad libitum food and water, and kept in a section of the refrigerator at 4 ± 2 °C for 14–15 h (overnight). All mice used in the behavioral experiments were then placed in the experimental room (20–23 °C) for at least 30 min for adaptation.

### Mechanical nociceptive test (von Frey filament test)

In the mechanical nociceptive test, mice were genotypically divided into two groups (*n* = 10): (1) WT group and (2) R222S group. The 50% mechanical threshold was determined using the von Frey filament test (Chaplan et al. 1994), using nine von Frey filaments with logarithmically incremental stiffness (0.008, 0.02, 0.04, 0.07, 0.16, 0.4, 0.6, 1.0, and 1.4 g). Each mouse was placed on a stainless-steel mesh floor covered with a plexiglass cage. The filament was pressed perpendicular to the plantar surface of the right hind paw to invoke a withdrawal response (biting, licking, lifting, or shaking). The baseline of 50% mechanical threshold was defined from mice at room temperature (20–23 °C). Subsequently, 50% mechanical thresholds were assessed at 0.5, 1, 1.5, 2, 3, 6, and 24 h after termination of cold exposure.

### Thermal nociceptive test (tail-flick test)

In the thermal nociceptive test, mice were genotypically divided into two group (*n* = 7): (1) WT group and (2) R222S group. Withdrawal latencies against thermal stimuli were measured using the tail-flick test with minor modifications (Garcia-Martinez et al. 2002; Tseng et al. 2011). Here, the light beam was focused 1–1.5 cm from the tail tip until flicking by using a tail-flick analgesiometer (Type7350, Ugo-Basile, Comerio, Italy). The intensity of the light beam was adjusted to a baseline level of between 3 and 5 s, which was defined from WT mice under room temperature (20–23 °C). The measurement for each mouse was repeated three times at intervals of 30 min. The average value of three measurements was taken as the withdrawal latency. The withdrawal latencies of cold-exposed mice were then measured within 1.5 h after termination of cold exposure.

### Measurement of pro-inflammatory mediators

WT and R222S mice were divided into four groups (*n* = 7–10): (1) WT naive group, (2) WT cold-exposed group, (3) R222S naive group, and (4) R222S cold-exposed group. In brief, mice were exposed to a cold atmosphere (4 ± 2 °C, overnight) and then euthanized under isoflurane anesthesia. Their blood and spinal cords were sampled within 2 h after cold exposure. Mice without cold exposure (naive mice; as a negative control) were also sacrificed and sampled as described above. Spinal cords were homogenized in RIPA buffer with a protease inhibitor cocktail and left for 30 min on ice, followed by centrifugation at 2000×*g* for 30 min. Whole-blood samples, obtained with a heparinized syringe, were transferred to a 1.5-mL tube and centrifuged immediately at 1700×*g* for 20 min. The separated supernatants and plasma samples were stored at − 80 °C prior to measurements. The levels of pro-inflammatory mediators, interleukin (IL)-1β, IL-6, tumor necrosis factor (TNF)-α, and interferon-γ, were measured using a cytokine assay system (Bio-Plex suspension Array System, Bio-Rad Laboratories) with the MILLIPLEX MAP Mouse Cytokine/Chemokine Magnetic Bead Panel - Immunology Multiplex Assay (Millipore), according to the manufacturer’s instructions.

### Measurement of the hind paw thickness

WT mice (*n* = 8) and R222S mice (*n* = 7) were anesthetized with isoflurane, and the thickness (mm, using digital micrometer calipers) of their right hind paw was measured at room temperature (20–23 °C). Subsequently, the right paw thicknesses of anesthetized mice were assessed within 2 h after termination of cold exposure.

### Treatments of analgesics

We subsequently examined whether mechanical allodynia in R222S mice could be a usable model to detect the antinociceptive effect of analgesics. AcAP was purchased from Nacalai Tesque Inc. (Kyoto, Japan). GJG was provided by Tsumura & Co. (Tokyo, Japan) as a spray-dried powder from a hot water extract of ten crude drugs in fixed proportions: *Rehmanniae* radix (5.0 g), *Achyranthis* radix (3.0 g), *Corni* fructus (3.0 g), *Moutan* cortex (3.0 g), *Alismatis* rhizoma (3.0 g), *Dioscoreae* rhizoma (3.0 g), *Plantaginis* semen (3.0 g), *Poria* (3.0 g), processed *Aconiti* tuber (1.0 g), and *Cinnamomi* cortex (1.0 g). Test drugs were freshly dissolved in distilled water (DW) prior to experiments.

According to the baseline of 50% mechanical thresholds, R222S mice were divided into different treatment groups (*n* = 8): a vehicle group, a low-dose drug group, and a high-dose drug group. The vehicle group of WT mice (*n* = 11–14) was also set as a negative control. After cold exposure, mice were evaluated for the 50% mechanical threshold and then orally administrated DW or the drugs: AcAP (125 or 250 mg/kg) or GJG (0.5 or 1.0 g/kg). The dose of AcAP and GJG used in the present study approximately paralleled the typical clinical daily dose and was converted based on the body surface area according to guidelines from the US Food and Drug Administration (available from URL https://www.fda.gov/regulatory-information/search-fda-guidance-documents/estimating-maximum-safe-starting-dose-initial-clinical-trials-therapeutics-adult-healthy-volunteers).

### Statistics

To evaluate the efficacy of the analgesics, the area under the curve (AUC, mg*h) for mechanical thresholds (mg) from 0 (before oral administration) to 3 h after administration was calculated. Data with error bars represent the means ± standard error of the mean (SEM). Multiple comparisons were performed by using one- or two-way analyses of variance (ANOVA) with post hoc Holm–Sidak’s test or Dunnett’s test using the Graphpad Prism version 7 software. *p* value < 0.05 was considered statistically significant.

## Results

### Mechanical allodynia and thermal hyperalgesia in R222S mice occurred after cold exposure

**Fig. 1:**
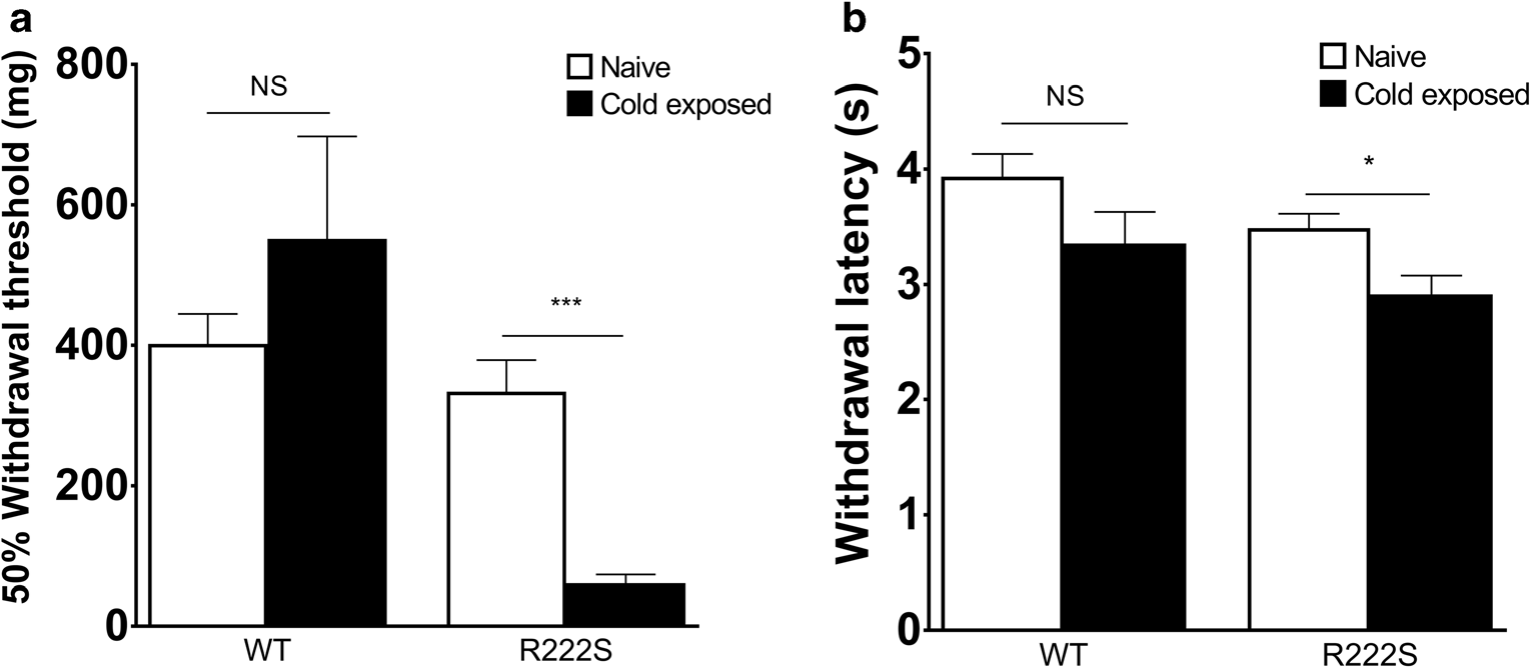
Influence of cold exposure on mechanical and thermal sensitivity in wild type and *Scn11a* p.R222S mutant mice. **a** The 50% mechanical withdrawal threshold (mg) in wild type (WT, *n* = 10) mice and *Scn11a* p.R222S mutant (R222S, *n* = 10) mice. **b** The thermal withdrawal latency (seconds) in WT (*n* = 7) mice and R222S (*n* = 7) mice. WT and R222S mutant mice were exposed to cold (4 °C, overnight), and the mechanical/thermal sensitivities within 1.5 h were measured. Naive, mice housed in room temperature (20–23 °C); Cold exposed, mice after cold exposure (4 °C, overnight). Data are presented as the mean ± SEM. Statistical significance was calculated using Welch’s *t* test. *p < 0.05, ***p < 0.001. NS, not significant

**Fig. 2:**
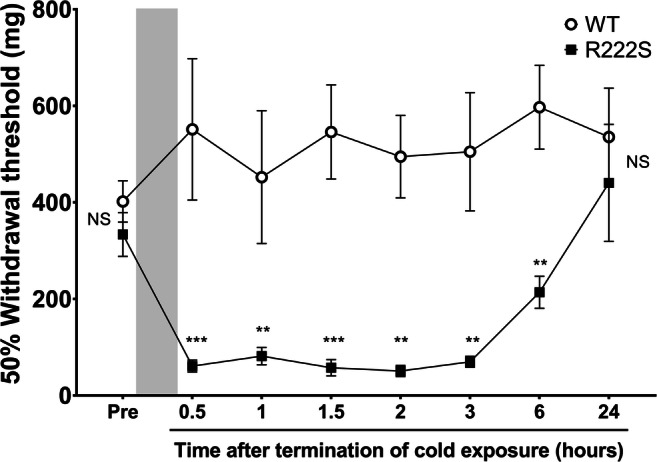
Time course of the 50% mechanical withdrawal threshold in cold-triggered wild type and *Scn11a* p.R222S mutant mice. Wild type (WT, *n* = 10) and *Scn11a* p.R222S mutant (R222S, *n* = 10) mice were exposed to cold (4 °C, overnight), as shown by the gray zone. The 50% mechanical withdrawal threshold was evaluated at 0.5, 1, 1.5, 2, 3, 6, and 24 h after termination of cold exposure. Data are presented as the mean ± SEM. One-way or two-way ANOVA followed by Holm–Sidak’s multiple comparison test was performed to compare WT vs R222S mice at each time point. **p < 0.01, ***p < 0.001. NS, not significant

To assess the mechanical/thermal withdrawal behavior in mice, we used the von Frey filament test and the tail-flick test, which are common test batteries for neuropathic pain models that can stimulate a diseased area intensively and noninvasively (Sandkuhler 2009). As shown in Fig. 1a, there was no significant difference in response to mechanical or thermal stimuli in WT mice after exposure to a cold atmosphere. The result of the von Frey filament test suggested that cold-exposed R222S mice exhibited remarkable mechanical allodynia of the hind paw. This mechanical hypersensitivity of the R222S mice was significant from 0.5 to 6 h after cold exposure and only reversed to WT levels by 24 h after housing at room temperature (Fig. 2). In the tail-flick test (Fig. 1b), we found that cold-exposed R222S mice also exhibited significant thermal hypersensitivity, but it was relatively moderate compared with the change in mechanical sensitivity. We could not observe other behavioral changes, such as locomotor activity or diarrhea, in mice after cold exposure.

### No significant changes in biochemical or structural parameters in WT and R222S mice during cold exposure

**Fig. 3:**
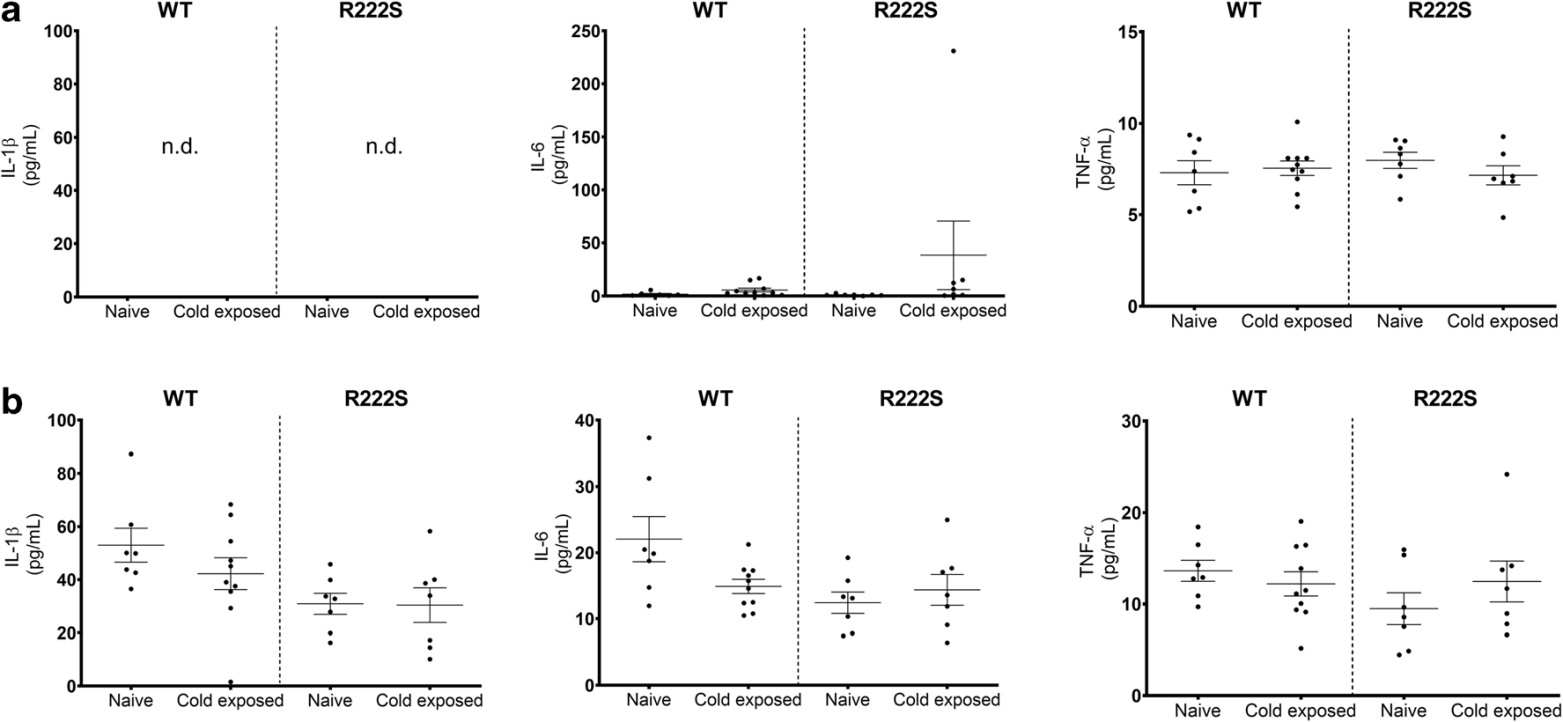
Pro-inflammatory mediator levels in plasma and spinal cord samples in wild type and *Scn11a* p.R222S mice. The levels of pro-inflammatory mediators in plasma (**a**) and spinal cord (**b**) samples were determined using a cytokine assay system (Bio-Plex suspension Array System, Bio-Rad Laboratories). Wild type (WT) and *Scn11a* p.R222S mutant (R222S) mice were divided into four groups (*n* = 7–10/group): (1) WT naive group, (2) WT cold-exposed group, (3) R222S naive group, and (4) R222S cold-exposed group. Naive, mice housed in room temperature (20–23 °C); Cold exposed, mice after cold exposure (4 °C, overnight). n.d., not detected. Data are presented as the mean ± SEM

To assess the involvement of inflammatory signals in the behavioral changes of R222S mice during cold exposure, we analyzed the changes in pro-inflammatory mediators in the plasma and spinal cords of the test mice (Fig. 3). Compared with the naive mice, the levels of IL-1β, IL-6, and TNF-α did not significantly differ between WT and R222S mice in response to cold exposure. We also measured the levels of interferon-γ, but these were undetectable in both the plasma and spinal cord samples. We subsequently measured the hind paw thickness of WT and R222S mice to examine whether cold exposure induced peripheral edema. However, as shown in Table 1, there were no significant differences between the mice.

**Table 1:** Changes in hind paw thickness in wild type and *SCN11A* p.R222S mice following cold exposure

	Naive	Cold exposed
WT	2.72 ± 0.03 mm	2.66 ± 0.02 mm
R222S	2.70 ± 0.02 mm	2.69 ± 0.03 mm

No edematous changes in the hind paws of wild type (WT, *n* = 8) and *Scn11a* p.R222S (R222S, *n* = 7) mice were induced after cold exposure. Mice were anesthetized with isoflurane, and the thickness (mm, using digital micrometer calipers) of the right hind paw was measured. Naive, mice prior to cold exposure (4 °C, overnight); cold exposed, mice after cold exposure. Data are presented as the mean ± SEM per group

### GJG and AcAP significantly attenuated mechanical allodynia in R222S mice

**Fig. 4:**
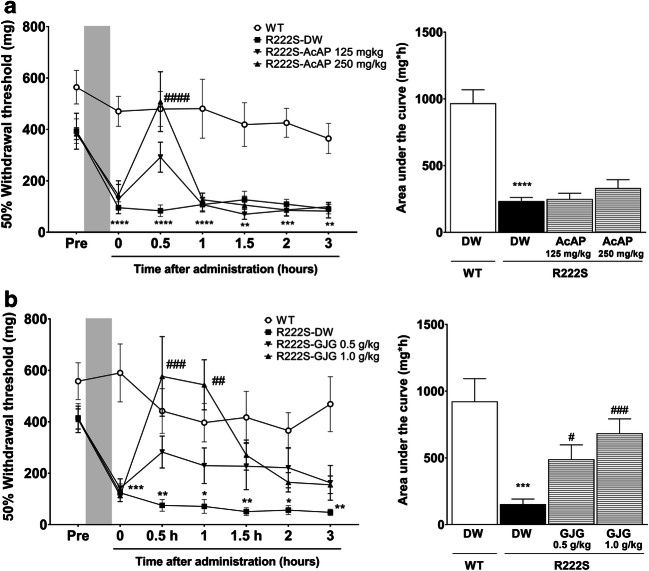
Therapeutic effects of acetaminophen and goshajinkigan on the mechanical allodynia triggered by cold exposure. Wild type (WT, *n* = 11–14) or *Scn11a* p.R222S mutant (R222S, *n* = 8/group) mice were orally administrated DW vehicle or drugs. **a**, **b** Time course of acetaminophen (AcAP) or goshajinkigan (GJG) effects on cold-triggered mechanical allodynia in R222S mice and area under the curve (AUC). Data are presented as the mean ± SEM. Two-way ANOVA followed by Holm–Sidak’s multiple comparison test was used to calculate the significance of each time course. *p < 0.05, **p < 0.01, ***p < 0.001, ****p < 0.0001 vs the WT DW group, ^##^*p* < 0.01, ^###^*p* < 0.001, ^####^*p* < 0.0001 vs the R222S DW group. Statistical significance in AUC was calculated by Welch’s *t* test ***p < 0.001, ****p < 0.0001 vs the WT DW group and by one-way ANOVA and post hoc Dunnett’s test ^#^*p* < 0.05, ^###^*p* < 0.001 vs the R222S DW group

Finally, we evaluated the antinociceptive effects of GJG and AcAP in the model of cold-triggered mechanical allodynia, using orally administered DW as vehicle control. As shown in Fig. 4, the mechanical thresholds of WT mice treated with cold exposure remained unchanged for 3 h after DW administration. In contrast, R222S mice given DW (R222S-DW control) clearly displayed cold-triggered mechanical allodynia. The administration of high-dose AcAP (250 mg/kg) significantly improved mechanical allodynia of R222S mice at 0.5 h (*p* < 0.0001) relative to the R222S DW control; however, its effect rapidly disappeared before 1 h (Fig. 4a). The administration of high-dose GJG (1.0 g/kg) attenuated the mechanical allodynia of R222S mice at the 0.5- (*p* < 0.001) and 1- (*p* < 0.01) h time points in comparison with the R222S DW controls, but there were no significant differences after 1 h of GJG administration (Fig. 4b). To assess the intensity and duration of the antinociceptive effects of GJG and AcAP, we evaluated the AUC calculated from the mechanical thresholds (mg) from 0 (before oral administration) to 3 h after administration. GJG, but not AcAP, significantly increased the AUC compared with the R222S DW control group (Fig. 4a and b, respectively).

## Discussion

One of the major findings of this study is that mice with the *Scn11a* p.R222S missense mutation exhibited mechanical allodynia and thermal hypersensitivity triggered by cold exposure (Figs. 1 and 2). These cold-evoked pain behaviors of R222S mice were similar to the clinical symptoms of patients with familial episodic limb pain. There were no significant differences in the contents of inflammatory cytokines or in the swelling of the hind paw between WT and R222S mice during cold exposure (Fig. 3 and Table 1), implying that inflammatory symptoms were not involved in the behavioral changes of R222S mice in this study.

The hyperexcitability of sensory neurons may contribute to enhanced pain transmission (Bennett et al. 2019). Our previous study showed that the firing frequency with current injection in DRG neurons of R222S mice was higher than that of WT mice (Okuda et al. 2016). It is possible that the phenotypical changes in Na_V_1.9, resulting from the specific *SCN11A* missense mutation, are involved in the pathological mechanisms of hypersensitivity of the R222S mice. The level of Na_V_1.9 sodium current in small DRG neurons increases in the presence of inflammatory mediators (Maingret et al. 2008). In addition, posttranslational modifications of Na_V_ channels triggered by inflammatory mediators result in changes of firing properties in sensory neurons (Laedermann et al. 2015); however, neither biochemical nor structural evidence of inflammation was detected in this study. Cold exposure may alter other signaling events in the DRG, such as the overall profile of protein kinase, the specific upregulation of Fyn kinase that regulates Na_V_1.7 channels in response to repeated cold stress (Kozaki et al. 2015; Li et al. 2018), or even other posttranslational modifications, including phosphorylation, that can modify the trafficking and functions of Na_V_ channels in neurons (Laedermann et al. 2015). As suggested by previous clinical reports, the relationship of *SCN11A* gene mutations and environmental changes may be involved in painful disorders (Leipold et al. 2013; Zhang et al. 2013; Huang et al. 2014; Han et al. 2015; Woods et al. 2015; Okuda et al. 2016; Han et al. 2017; King et al. 2017; Castoro et al. 2018; Kabata et al. 2018; Huang et al. 2019). Clearly, further research, for example with stable Na_V_1.9-expressing cell lines, is necessary to clarify the molecular mechanisms of *SCN11A* gain-of-function mutations.

In the present study, mechanical allodynia in R222S mice provided a sufficient window to evaluate the antinociceptive effects of drug candidates. We considered the von Frey filament test to be an appropriate means for repeated, non-invasive measurements during short durations. The therapeutic administration of 250-mg/kg AcAP or 1.0-g/kg GJG significantly attenuated the mechanical allodynia in R222S mice after cold exposure (Fig. 4). This is thus the first report that clearly demonstrates that mechanical allodynia in R222S mice triggered by cold exposure can serve as an invaluable model to investigate therapeutic drugs for pain disorders related to the *SCN11A* gene mutation. The efficacy of AcAP found in the present study is in agreement with previous clinical reports (Okuda et al. 2016; Kabata et al. 2018), and a time course of its antinociceptive effects on mechanical allodynia in R222S mice (peak at 0.5 h after administration) is also consistent with a previous report (Mallet et al. 2008). AcAP is broadly prescribed as an antinociceptive drug for painful disorders, including familial episodic limb pain (Okuda et al. 2016; Kabata et al. 2018). The active metabolites of AcAP, N-acetyl-p-benzoquinone imine and p-benzoquinone, reduce voltage-gated calcium and sodium currents in primary sensory neurons through the activation of spinal TRPA1 (Andersson et al. 2011). In addition, AcAP and its other metabolites (p-aminophenol and AM404) produce the antinociceptive effect through multiple action mechanisms, including the inhibition of prostaglandin synthesis and reinforcement of the activities of the endocannabinoid system in the brain and serotonergic descending pain inhibitory pathway (Mallet et al. 2008; Graham et al. 2013; Jozwiak-Bebenista and Nowak 2014). Taken together, it is possible that AcAP attenuated mechanical allodynia in R222S mice through spinal and supraspinal analgesic mechanisms.

Interestingly, GJG was efficacious in the mechanical allodynia of R222S mice and may be indicative of its potential as a therapeutic candidate for *SCN11A* mutation–associated neuropathies. The analgesic effect of GJG on neuropathic pain is via upregulation of the spinal κ-opioid system (Suzuki et al. 1999a; Bahar et al. 2013; Higuchi et al. 2015; Toume et al. 2019). Aconite alkaloids in GJG are immediately absorbed into the rat plasma after oral administration (Kono et al. 2015) and contribute to the antinociceptive effects by suppressing the hyperexcitability of sensory neurons via persistent depolarization (Ameri 1998; Ren et al. 2012). In Fig. 4b, GJG attenuated mechanical allodynia in R222S mice, peaking at 0.5–1 h after administration. The time course of GJG analgesia in the present study corresponds to the duration of the analgesic effect and the increased peripheral blood flow found in diabetes models (Suzuki et al. 1998b, 1999a, 1999b). Recent studies suggest that improvements in peripheral blood flow by vasodilators also contribute to the alleviation of neuropathic pain (Gauchan et al. 2009; Ishida et al. 2019). *Alismatis* rhizome and *Dioscoreae* rhizome, which are components of GJG, also contribute to improved blood flow through inhibition of platelet aggregation (Suzuki et al. 1998a). As demonstrated by previous reports (Okuda et al. 2016; Kabata et al. 2018), warming of the lesions is effective in relieving pain in patients with familial episodic limb pain. Hence, the multiple mechanisms of GJG, including improvements of peripheral blood flow that contributes to lesion warming, may be involved in the analgesic efficacy of GJG on mechanical allodynia in R222S mice.

## Conclusion

This is the first report that mechanical allodynia and thermal hypersensitivity in mice carrying the *Scn11a* p.R222S missense mutation can be induced by cold exposure. Furthermore, the analgesic drugs, AcAP and GJG, were found to improve mechanical allodynia in R222S mice. We conclude that mechanical allodynia induced in R222S mice is suitable for evaluating the antinociceptive effects of these two drugs and that this novel pain model will be an invaluable tool for drug therapy research that targets *SCN11A*-related painful symptoms.

## Electronic supplementary material

ESM 1 (XLSX 37 kb)
